# Health-Related Quality of Life in Adult Patients with Strabismus—Translation and Psychometric Testing of the Adult Strabismus Questionnaire (AS-20) into Finnish

**DOI:** 10.3390/ijerph20042830

**Published:** 2023-02-06

**Authors:** Anna Mason, Katja Joronen, Laura Lindberg, Anna-Maija Koivisto, Nina Fagerholm, Anja Rantanen

**Affiliations:** 1 Faculty of Social Science, Health Sciences, Tampere University, 33014 Tampere, Finland; 2 Helsinki University Hospital, Helsinki University, 00290 Helsinki, Finland; 3 Department of Nursing Science, University of Turku, 20014 Turku, Finland

**Keywords:** adult strabismus questionnaire AS-20, health-related quality of life, ophthalmic nursing, strabismus, instrument validation

## Abstract

(1) Strabismus has an impact on individuals’ health-related quality of life. The impact should be assessed with valid patient-reported outcome measures such as the Adult Strabismus Questionnaire (AS-20). The AS-20 was further refined using a Rasch analysis for the American population. The aims of the study were to translate and culturally adapt the AS-20 into Finnish and to evaluate the psychometric properties of the Finnish AS-20. (2) The guidelines of the Professional Society for Health Economics and Outcomes Research steered the process and four items were added from the original data as Finnish additions. The construct and convergent validity and internal consistency were evaluated via psychometric testing for three potential Finnish AS-20 structures. The Strengthening the Reporting of Observational Studies in Epidemiology (STROBE) checklist was applied. (3) The participants (*n* = 137) reported that the translation was clear and understandable. All structures showed high reliability and internal consistency as measured using Cronbach alpha values. The convergent validity assessed using Spearman’s correlation coefficients between the structures and one item of Satisfaction with Life Scale indicated very low to moderate positive correlations. The construct validity evaluated using a confirmatory factor analysis revealed the refined AS-20 structure to be satisfactory. (4) The refined AS-20 can be used in clinical practice and research, but further validation is recommended.

## 1. Introduction

Strabismus is an ophthalmic condition where the eyes do not point in the same direction and a person struggles to focus their eyes on the same point. The eyes might deviate inwards, outwards, upwards or downwards. The eyes might also rotate inwards or outwards. This condition can also alternate in different directions or be intermittent, visible (tropia) or non-visible (phoria). Strabismus is present in all age groups, and approximately four percent of the adult population globally have this ocular condition [1,2]. Four percent equates to over 200 million adults globally when considering the 2019 United Nations Population Prospects for over 20-year-olds, so strabismus is not a minor ocular condition [3].

Strabismus impacts health-related quality of life (HRQOL) both functionally and psychosocially [4,5,6,7]. Everyday physical functioning may be impacted through diplopia (double vision), visual confusion, eye fatigue (asthenopia) and a lack of depth perception. Therefore, driving, working, general functioning and hobbies requiring clear sight might be difficult [4]. Psychosocially, individuals might have trouble in interactions and interpersonal relationships or feel general embarrassment due to the condition. Feelings of social phobia, anxiety, avoidance and depression have also been reported [7,8,9]. Complaints of ocular pain due to strabismus are common, and the condition can cause head, back and neck pain due to assuming awkward head positions for better vision [4,5,6]. All of these factors impact an individual’s HRQOL, which consists of mental, physical and social well-being [10]. 

HRQOL among strabismic adults can be evaluated with generic quality of life measures, but those are not sensitive enough to assess functional struggles with vision or psychosocial challenges that individuals with strabismus exhibit. Therefore, condition-specific instruments are recommended [11].

The AS-20 was developed in the USA and is based on qualitative interviews on the influence of strabismus on an individual’s HRQOL [2]. The interviews generated 181 items that were piloted on adult strabismus patients. Items with low or high response rates or a focus purely on symptoms were removed. Item bias was considered by removing items possibly discriminative to some patients (i.e., driving or economic status). The original AS-20 has 20 items divided into psychosocial (items 1–10) and functional (items 11–20) subscales, and HRQOL is measured using a 5-point Likert scale (never, rarely, sometimes, often and always). Individuals choose the option which best reflects the impact of strabismus on their HRQOL, and the responses are calculated as points, where never scores 100, rarely 75, sometimes 50, often 25 and always 0 points. The overall value of HRQOL is calculated by the mean sum of completed items, and a low overall score indicates low HRQOL [2]. Median scores (Md) of visually normal adults have been reported higher with overall (Md 98), psychosocial (Md 100) and functional (Md 95) HRQOL compared to strabismic adults (Md 56, Md 73 and Md 40, respectively) [2].

The original AS-20 is a valid and sensitive tool with good discriminative validity to measure HRQOL for strabismic adults, and its’ internal consistency, on the whole measure and for both subscales, has been proven to be high by its Cronbach alfa >0.90 [2]. The AS-20 is also responsive to changes in HRQOL, such as after strabismus surgery, and has good test–re-test ability [12,13].

The original AS-20 was further refined by a Rasch analysis indicating that four subscales rather than two were more accurate to assess HRQOL in the American population. Two items from the functional subscale (items 14 and 19) are not scored in the refined AS-20. A patient’s HRQOL is assessed using subscales of self-perception (items 1–4, 6), interaction (items 5, 7–10), reading function (items 12–13, 16, 20) and general function (items 11, 15, 17–18). Additionally, response options of never and rarely are combined in the scoring of the general function subscale. The HRQOL is calculated using the provided look-up table or by computing the means of all completed items separately for each of the four subscales [14]. In the refined AS-20, the self-perception and the reading function subscales are reported to be reliable, whereas the reliability of the interaction and general function subscales is reported to be less than optimal [14]. 

The impacts of strabismus are seen in ophthalmic departments around the world. The treatments include surgical and non-surgical care options, such as prism glasses and orthoptic exercises [9]. In some countries with socialized healthcare, adults who suffer merely psychosocial impacts from strabismus might not receive surgical treatment, as the surgery is seen as a cosmetic procedure and is not available through the healthcare system [1]. The treatment should be available regardless of the impact of strabismus [1]. Therefore, patient-reported outcome measures should be utilized to aid in clinical decision-making and to improve care [15]. Nurses have a particular role in developing patients’ holistic care [16] and should be active in developing care processes for strabismus patients’ welfare.

To our knowledge, there has been no specific instrument available to measure HRQOL among Finnish adults with strabismus. Therefore, the aim of this study was to translate and culturally adapt the AS-20 into the Finnish language and culture, and to evaluate the psychometric properties and the descriptive statistics of the Finnish AS-20. 

## 2. Materials and Methods

### 2.1. Design

A cross-sectional prospective study was performed to (1) translate and culturally adapt the AS-20 into Finnish and (2) to evaluate the psychometric properties and the descriptive statistics of the Finnish AS-20. The Strengthening the Reporting of Observational Studies in Epidemiology (STROBE) guidelines steered the presentation of this study and the results (Supplementary File S1).

AS-20 is in the public domain. The original developers were contacted regarding the translation and validation process in 2019, and permission was received to use some of the original 181 items for cultural adaptation [17]. The original AS-20 does not have items that reflect common complaints of Finnish strabismic adults such as walking up and down steps, walking on uneven surfaces, playing sports or using mobile devices. Therefore, the authors (L.L., A.M.) chose potential items from the original 181 items [17] to reflect Finnish patients’ experiences and consulted a multi-professional team for their clinical experiences of strabismic patient care. Four items were chosen and confirmed by the rest of the research team (K.J., A.K., N.F., A.R.) as relevant to the Finnish culture and context. These four items were added as Finnish additions to the questionnaire for cultural adaptation. The items were: “I find it difficult to go up and down steps”, “I have problems walking on uneven surfaces” and “It is difficult for me to play sports because of my eyes” [17]. As our society is very dependent on smart devices and their use has increased since the original questionnaire was developed, an item was modified from “I have problems looking at a computer screen” [17] to “It’s difficult for me to use mobile devices because of my eyes”. These items are not specific to Finnish culture but are commonly reported by Finnish strabismic adults as functional impacts of strabismus. The use of the Finnish additions could enrich the AS-20 questionnaire in other clinical environments with similar socio-cultural contexts. 

### 2.2. Instruments

The demographic background questions included the year of birth, sex and highest level of education (comprehensive school, diploma, bachelor’s or master’s degree, licentiate or PhD). The strabismus-related background variables included the presence (one, both eyes or not sure) and visibility (yes/no) of the strabismus, impact of the strabismus on work (no; yes partly; yes fully; I am not working), tiredness of the eyes (yes/no), presence of diplopia (yes/no/not sure), need for near vision for work or hobbies (yes/no) and number of strabismus surgeries.

Global life satisfaction was measured by one item of the Satisfaction with Life Scale (SWLS), “I am satisfied with my life”. The Finnish SWLS uses a 5-point Likert scale, as the original English SWLS contains response options that are too close in the Finnish language. In the Finnish SWLS, the response options are “fully disagree, partly disagree, neither disagree nor agree, partly agree and fully agree” [18,19]. 

The first author (A.M.) contacted the developers of the AS-20 [2] to understand the scoring and structure of the AS-20, and the use of a refined AS-20 was recommended. As it was not known how the AS-20 performs in the Finnish population, it was important also to evaluate the psychometric properties of the original valid AS-20 structure. Gothwal et al. [20], in their translation and validation of the AS-20 in India, recommended that the validity of the AS-20 should be evaluated prior to making changes regarding the subscales of the AS-20, as strabismic populations vary in their clinical and background characteristics. Additionally, as the original AS-20 was missing culturally important items, four Finnish additional items were added as described here. Therefore, this study assessed three potential structures and scoring for the Finnish AS-20, which were the original AS-20, the original AS-20 with Finnish additions items and the refined AS-20. To our knowledge, the inclusion of the items from the original data has not been studied before.

### 2.3. Translation Process

The translation and cultural adaptation process followed the guidelines recommended by The Professional Society for Health Economics and Outcomes Research [21]. All items were first forward-translated into Finnish by two researchers (A.M., L.L.) and the translation was assessed by the research group, including researchers from the fields of nursing science, statistics and medicine. All members of the research group were native Finnish speakers and fluent in the English language. During the discussions, some minor changes were conducted, such as for item 5 “People do not give me opportunities because of my eyes” an explanation of “for example in the working life” was added, and for the item 16 “I have problems reading because of my eye condition” was changed from “Due to my eye condition it is difficult for me to read” to “The state of my eyes makes reading more difficult”. After the changes, an agreement was reached on the forward translation and the first Finnish version of the AS-20 was approved. It was then sent to five native Finnish speaking adults for cognitive debriefing and comments were requested on the clarity and fluency of the forward translation [21]. They reported the translation to be clear and comprehensible, but a few corrections were suggested regarding Finnish grammar and re-wording, changing item 10 from “I find it hard to initiate contact with people I don’t know because of my eyes” to “Because of my eyes it’s hard for me to take the initiative or to make contact with people I don’t know”. The corrections were discussed in the research team and agreed upon and the instrument was changed accordingly. 

Next, the Finnish AS-20 was back-translated into English by a professional interpreter and afterwards compared to the original English AS-20 by the research team. Even though all items were not literally identical, the meaning was the same. Therefore, the Finnish version of AS-20 was approved by the research group for pilot testing. 

### 2.4. Participants and Data Collection

This study was conducted in a university hospital’s ophthalmology clinic in Finland caring for people with strabismus. In 2019, there were 3400 strabismus outpatient visits and nearly 350 strabismus surgeries performed. The data collection began in December 2019 and finished in December 2020. The COVID pandemic slowed the data collection as the number of patients attending the outpatient clinic was reduced due to the pandemic.

There are no universally accepted guidelines for sample sizes for validation studies, and the minimum numbers vary [22]. In this study, the sample size was calculated as requiring 5 subjects per item to achieve a minimum of 120 participants [23]; therefore, a total of 150 participants with visible strabismus or symptomatic phoric patients were recruited to prepare for possible losses in participation. The criteria for participation were the following: adult (18+ years) patients in the ophthalmology clinic, fluent in Finnish, without cognitive impairments or critical conditions affecting HRQOL. As the aim was to validate an instrument measuring HRQOL, participants with severe conditions, including patients with thyroid eye disease, heart or brain infarcts and other severe somatic or psychiatric illnesses were excluded. Patients who had received recent surgery were also excluded, as side effects from the surgery could have affected their HRQOL. To achieve the limitation, two researchers (A.M., L.L.) marked in advance potential patients coming to the clinic so that the staff could offer them purposeful participation in the study. The patients were given an information letter regarding the study on arrival and the staff checked the participation criteria. The patients were told that participation was voluntary and would not alter their care. Written consent was obtained from all participants.

A total of 150 participants consented to the study. They were given a postage paid envelope that contained a questionnaire with background questions and the translated AS-20 with Finnish additions. They could fill in the questionnaire independently in the clinic or at home, and could then return it to the researchers. Questionnaires were returned by 138 patients. One of the participants was found to have a severe long-term illness, so one questionnaire was left out of the analysis. The data for a total of 137 participants were analyzed.

### 2.5. Data Analysis 

The data on the participants’ demographic and strabismus-related variables, satisfaction with life and HRQOL sum scales were described using frequencies and percentages for qualitative variables and the mean (M), standard deviation (SD), median (Md) and quartiles (Q1, Q3) for quantitative variables. Participant responses for the original AS-20 and AS-20 with Finnish additions were calculated as points, whereby never scored 100 points, rarely 75, sometimes 50, often 25 and always 0 points [2]. For the refined AS-20 structure, the descriptive statistics were calculated both by computing the mean of all completed items separately for each four subscales (clinical use) and by using the provided look-up table [14]. This was to compare whether there was a difference in Finnish participants’ mean sums when the scores were calculated using either a 5-point scale (clinical use, options of never, rarely, sometimes, often, always) or a 4-point scale (look-up table), where in the general function subscale “never” and “rarely” are combined as one option. 

All statistical analyses evaluating the psychometric properties of the Finnish AS-20 were conducted on three different structures: (1) for the whole original AS-20 measure and its’ two subscales; (2) for the original AS-20 measure with Finnish additions; (3) for the refined AS-20 with four subscales. The psychometric properties evaluated were the internal consistency, construct validity and convergent validity. The data were analyzed using IBM SPSS Statistics version 25 and a confirmatory factor analysis (CFA) was performed on MPlus 8.5. Statistical significance was set at *p* < 0.05 [24].

#### 2.5.1. Internal Consistency

The internal consistency describes how well the items of an instrument evaluate the same construct. In this study, internal consistency was calculated using Cronbach alfa values (α), as all items on the AS-20 evaluate HRQOL. The calculations were conducted separately on overall scores for the AS-20 and AS-20 with Finnish additions and their subscale scores, and for the refined AS-20 subscales. The lowest acceptable value was set to be 0.70 [25]. 

#### 2.5.2. Convergent Validity

The convergent validity is assessed by testing if the instrument used for validation has an association with another instrument measuring similar constructs. As there are no validated measures to evaluate HRQOL among Finnish strabismic adults, the convergent validity was tested by calculating Spearman correlation coefficients between one item (“I am satisfied with life”) of the Satisfaction with Life Scale (SWLS) with overall scores for the AS-20 and AS-20 with Finnish additions and their subscale scores, and for the refined AS-20 subscales. Spearman’s correlation coefficient was selected because the items from the SWLS scale were of an ordinal scale and the distributions of the sums of AS-20 subscales were skewed. Values of 0.20−0.49 were defined as having a low correlation, 0.50−0.69 as moderate, 0.70−0.89 as high and 0.90−1.00 as very high [26]. 

#### 2.5.3. Construct Validity

The construct validity is the degree to which the evaluated instrument measures the studied construct and how the instrument provides scores based on previous knowledge or theory. In this study, the CFA was used to assess the construct validity, as the English AS-20 was already shown to validly assess HRQOL among strabismic adults [25,26,27]. Different fit indices were examined to assess the fit of the three potential Finnish structures of the AS-20, including the chi-square goodness of fit, root mean square error of approximation (RMSEA), standardized root mean square residual (SRMR), Comparative Fit Index (CFI) and Tucker–Lewis Index (TLI). Cut-off values close to 0.95 were used for the CFI and TLI, 0.08 for the SRMR and 0.06 for the RMSEA [27,28]. A non-significant chi-square value was an indication of a good fit [29]. The CFA models for the three Finnish structures of the AS-20 were estimated using items as categorical variables with the weighted least square mean and variance-adjusted estimation method (WSLMV) [30].

### 2.6. Ethical Considerations

The Declaration of Helsinki and national guidelines for responsible research conduct were followed [31,32]. The ethical board of the healthcare organization approved the study and permission was granted. Participation in the study was voluntary and did not alter the care for the participating patients, who all gave written informed consent. The participants were informed that they could discontinue their participation at any stage of the study. Confidentiality was maintained and the organization’s data policy was followed.

## 3. Results 

### 3.1. Participants

All participants filled in the questionnaire independently, either in the clinic or at home, and posted it to the researchers. The response rate for this study was 91%. More than half of the study participants were women (59%, *n* = 81) and over three-quarters had either a vocational diploma or degree (78%, *n* = 107). The age range of the participants matched the age range of the adult patients in the clinic, as the youngest participant was 18 and the oldest were 84 years old (M = 47, SD = 17). One-third of the participants (30%, *n* = 41) reported not to be working.

Over half of the participants reported having strabismus purely on one eye (55%, *n* = 75) and over three-quarters (79%, *n* = 107) described their strabismus as being visible. Tiredness of the eyes was a very common symptom for the participants (89%, *n* = 122), and nearly two-thirds reported suffering from diplopia (63%, *n* = 86). Nearly all reported needing near vision for work or hobbies (97%, *n* = 133). Half of the patients (52%, *n* = 71) communicated that the strabismus had a partial influence on their work, but they managed in their own job. Regarding surgery, nearly half of the patients (44%, *n* = 60) had had at least one surgery. 

Table 1 presents the participants’ background variables and strabismus-related characteristics.

### 3.2. Translation and Adaptation Process

Most participants found the translated Finnish AS-20 items to be understandable or extremely understandable (91%, *n* = 124) and the clarity of the questionnaire was assessed by most as very or extremely clear (91%, *n* = 125). Some participants suggested adding more items on ocular pain, discomfort and tiredness (*n* = 11); eye glass usage (*n* = 5), driving (*n* = 5); and psychosocial concerns, particularly regarding pressure related to appearance and social interactions (*n* = 8). The participants (*n* = 8) criticized the response option “never” for items 5 and 19, as this was a double-negative, and recommended changing “never” into “not applicable” or changing the items into positive statements. Additionally, four participants commented on item 14 being unclear and recommended providing an example of depth perception for the item. The participants also expressed joy in the questionnaire (*n* = 5) that strabismus and its impact on HRQOL is studied in Finnish settings. 

The participants’ responses to the AS-20 items varied from never to always, although the option “always” was the least used. Regarding the interactions, it is noteworthy that over half of the participants chose “never” for items 7, 9 and 10 (53%, *n* = 72; 54%, *n* = 74; 56%, *n* = 76, respectively), and three-quarters of patients selected “never” for item 5 (75%, *n* = 100). Table 2 shows the AS-20 and Finnish additional items and their frequencies and percentages. 

The functional HRQOL of the participants was lower (Md = 53, Q1 = 38, Q3 = 66) than the psychosocial HRQOL with the original AS-20 structure (Md = 75, Q1 = 54, Q3 = 90), whereas with the refined AS-20, the participants’ HRQOL based on the interaction subscale was higher (Md = 88, Q1 = 65, Q3 = 100) than the self-perception subscale (Md = 60, Q1 = 38, Q3 = 85). For the refined AS-20, the sum scores were similar for the three subscales, despite the method of calculation that was used. However, for the general function subscale, the participants’ sum scores were much lower with the clinical use calculation method in comparison to the look-up table (Md = 44, Q1 = 34, Q3 = 59 vs. Md = 60, Q1 = 47, Q3 = 76, respectively). 

Table 3 presents descriptive the statistics and psychometric properties of all three Finnish AS-20 questionnaire structures and their subscales.

### 3.3. Internal Consistency 

The reliability and internal consistency were analyzed separately using the Cronbach alphas for the AS-20 original and AS-20 with Finnish additions, their subscales and the refined AS-20 subscales. The results showed that the overall scores for the original AS-20 and AS-20 with Finnish additions showed high internal consistency and reliability (α = 0.919, 0.923, respectively). Furthermore, the subscales of the refined AS-20 had strong Cronbach alpha values, except for the general function subscale, which was borderline (α = 0.675). 

### 3.4. Convergent Validity

Over three-quarters of the study participants were either partly (40%, *n* = 55) or fully (37%, *n* = 46) satisfied with their life, as measured by the item “I am satisfied with my life”. Spearman’s correlation coefficient showed very low to moderate positive correlations between the item and the overall scores of the original AS-20 and AS-20 with Finnish additions (*r* = 0.501, 0.474, respectively). The correlation coefficient for the refined AS-20 was moderate for the self-perception (*r* = 0.439) and interaction subscales (*r* = 0.456), but there was a very low positive correlation between “I am satisfied with my life” and the sum scales of the reading function subscale (*r* = 0.194). 

### 3.5. Construct Validity

A CFA was conducted on the three structures of the Finnish AS-20. In the CFA of the original AS-20 and the original with Finnish additions, 131 participants were included, whereas in the CFA of the refined AS-20, 132 participants were included. The chi-square goodness of fit was statistically significant, indicating no fit for all structures, and the RMSEA values were higher than the recommended cut-off value of 0.06 for all structures. The SRMR values were higher than the recommended value of 0.08 for the original AS-20 and original AS-20 with Finnish additions (0.124 and 0.121, respectively), whereas the value for the refined AS-20 structure was acceptable (0.077). The CFI and TLI values for all structures were within the recommended cut-off value range. The model fit indicators for the three structures of the Finnish AS-20 are presented in Table 4.

## 4. Discussion

This study aimed to translate and culturally adapt the Adult Strabismus Questionnaire (AS-20) to the Finnish language and culture and to evaluate the psychometric properties and descriptive statistics of the Finnish AS-20. The AS-20 was chosen for validation, as it is a specific measure used for evaluating HRQOL among strabismic adults [2,12]. 

### 4.1. Translation and Adaptation Process

The translation process proceeded according to Wild et al. [21]. The developers of the AS-20 were contacted for permission for translation, but also to understand the scoring of the measure to be able to keep the translated AS-20 as close to the original as possible [25]. However, as the measure also needs to be valid culturally [21,25], four additional items, the Finnish additions, were chosen from the original AS-20 development data [17] to present the challenges faced by Finnish strabismic adults. This improved the cultural adaptation, as the measures are aimed toward the target population [25]. 

The participants in the study were Finnish-speaking adults with strabismus who had attended an outpatient clinic in the search for help for their condition. Purposive sampling with inclusion criteria was used to increase the validity of the measures in the Finnish language and to decrease the risk for other factors than strabismus influencing the HRQOL. The participants for the study reflected the target population the AS-20 is designed for [25]. 

The participants’ comments on the double-negativity on items 5 and 19 were noted. As item 5 also showed very little variation in the participants’ response options (75% replied never), the research team considered whether this was due to the item’s wording. A decision was made to re-word item 5 from a negative to a positive statement “Because of my eyes my opportunities (for example in the working life) are reduced”. As the items 14 and 19 showed greater variation in the responses, the research team decided not to change the wording. For item 14, only four participants mentioned difficulty with the item. It is noteworthy that neither items 14 nor 19 are scored in the refined AS-20 [14]. 

Item 5 also needed revision in Chinese and Danish translations of the AS-20 [33,34]. In both studies, as in this study, participants commented on problems with driving as an additional factor impacting their HRQOL [33,34]. Item bias was considered in the AS-20 development phase by removing items potentially discriminative to some patients [2]; therefore, problems with driving or eye glass usage are not present in the Finnish AS-20 either. Additionally, the AS-20 aims to evaluate the impact of strabismus on HRQOL, not its symptoms [2]; hence, headache, neck or ocular pain and tiredness are not present in the Finnish AS-20. 

In the future, it will be important to assess whether the participants respond differently to item 5 after changing it into a positive statement. It might also be necessary to reduce the number of response options in the Finnish AS-20. However, some of the results regarding interactions or opportunities could be due to the Finnish society of equality and culture for social interactions. 

### 4.2. Psychometric Properties

The internal consistency as assessed by the Cronbach alpha values was high for both the Finnish original AS-20 and AS-20 with Finnish additions. The original AS-20 and its subscales and the Danish and Chinese translations of the instrument have been shown to have good internal consistency [2,33,34]. The refined structure of the Finnish AS-20 showed good internal consistency for three of its’ subscales, while the fourth, the general function, was slightly under the recommended cut-off value (Cronbach α = 0.675). Leske et al. [14] reported that interaction and general function subscales have less than optimal reliability. This was only seen for the general function and not the interaction subscale of the Finnish refined AS-20.

The convergent validity as assessed by the correlations between all sum scales of Finnish AS-20 structures and an item of the SWLS measure showed very low to moderate positive correlations. Although the item of SWLS does not directly measure HRQOL but instead measures satisfaction with life, it is noteworthy that the correlations were positive. As there are no translated and validated HRQOL measures for strabismic or ophthalmic patients, the SWLS was chosen. It is also possible that the use of the Finnish SWLS, with a five-point Likert scale rather than a seven-point scale, influenced the results [18,19].

The construct validity values of the three Finnish AS-20 structures were evaluated using the CFA, and the sample size met the recommendations for CFAs [29]. The chi-square values were significant, as were the non-fit model values for all structures, although Tabachnick and Fidell [27] (p. 770) stated that the use of small or large sample sizes might affect the chi-square values. Additionally, if the sample size is small (<250), Hu and Bentler [28] recommend focusing on a combination of the SRMR and CFI values to minimize the error rates. Based on the combination of these two values, the refined AS-20 structure with 18 items and four subscales showed acceptable construct validity for the Finnish AS-20. 

Leske et al. [14] recommend the refined AS-20 for research and clinical work. Although they advise using a look-up table to score the measure, it is important to note the difference in the Finnish participants’ scores for the general function subscale between the clinical use calculations, where the items were graded using a five-point Likert scale, and the look-up table calculations, where the options of never and rarely were combined. In future research, the method used for score calculations should be considered. As this study has piloted the Finnish additions with the original AS-20 and validates the Finnish AS-20 for only this specific sample and situation, it is necessary to continue the validation process for the Finnish AS-20 [25]. 

### 4.3. Strengths and Limitations

The translation of the Finnish AS-20 items was reported to be understandable, clear and showed high acceptability by the participants. The high response rate (91%) shows that studying the impact the strabismus has on HRQOL is also necessary in the Finnish context, and using the patient-reported outcome measure it can be validly measured. These increase the strength of this study.

For cultural adaptation, four additional items were chosen from the raw data [17]. The Finnish additions make the AS-20 more applicable to the target group and improve the clinical use of the instrument [25] and the strength of this study. Although in this study the participants used all response options for the Finnish additional items, the structure of the AS-20 with Finnish additions requires further studying and larger samples to assess whether the experiences of Finnish clinicians can also be seen in wider research results. 

There are limitations in this study. Although all Finnish AS-20 structures showed good internal consistency and fair convergent validity, the construct validity was acceptable only for the refined AS-20. This could have been due to the missed responses, the translation of the items or the small sample size used for the CFA. Even though the participants represented the patients in the outpatient clinic well, the sample size was small. As purposive sampling was used to avoid other illnesses than strabismus influencing HRQOL, it is possible that some important patient experiences were missed or highlighted. 

## 5. Conclusions

We have provided three structures for the Finnish AS-20 to evaluate the HRQOL among adults with strabismus. In this study sample, the refined AS-20 proved to be the most satisfactory structure for the Finnish AS-20. The Finnish AS-20 is an understandable and clear HRQOL measure for clinical use for Finnish strabismic patients to improve their care. Additional research in this area is recommended to further validate the Finnish AS-20 structure, its clinical utility and the scoring for research use. 

## Figures and Tables

**Table 1:** The participants’ self-reported characteristics, *n* = 137.

Background Variables	*n*	%
Sex		
Male	56	40.9
Female	81	59.1
Age		
18–30	24	17.5
31–44	44	32.1
45–63	41	29.9
64–84	28	20.4
Highest education		
Comprehensive	23	16.9
Diploma	44	32.4
Degree	63	46.3
Licentiate or PhD	6	4.4
Strabismus related variables		
Presence of strabismus		
One eye	75	54.7
Both eyes	55	40.1
Not sure	6	4.4
Is strabismus visible		
Yes	107	78.7
No	29	21.3
Tiredness of eyes		
Yes	122	89.1
No	15	10.9
Double vision (diplopia)		
Yes	86	62.8
No	51	37.2
Do you need near vision for work/hobbies		
Yes	133	97.1
No	4	2.9
Does strabismus effect work		
No	22	16.1
Yes partly, doing own role	71	51.8
Yes fully, unable to perform in my own role	3	2.2
Not working currently	41	29.9
Previous strabismus surgery		
None	77	56.2
One	44	32.1
Two	12	8.8
Three or more	4	2.9

**Table 2:** Frequencies and percentages of participants’ responses to the translated Adult Strabismus Questionnaire (AS-20) and Finnish additional items (*n* = 137; ^†^ Hatt et al. [2], Hatt et al. [17]).

	Never		Rarely		Sometimes		Often		Always	
	*n*	%	*n*	%	*n*	%	*n*	%	*n*	%
AS-20 Psychosocial Subscale										
1. I worry about what people will think about my eyes	35	25.5	26	19.0	37	27.0	27	19.7	12	8.8
2. I feel that people are thinking about my eyes when they do not say anything	43	31.4	26	19.0	37	27.0	24	17.5	7	5.1
3. I feel uncomfortable when people are looking at me because of my eyes	40	29.2	23	16.8	42	30.7	21	15.3	11	8.0
4. I wonder what people are thinking when they are looking at me because of my eyes	45	32.8	33	24.1	26	19.0	25	18.2	8	5.8
5. People do not give me opportunities because of my eyes, for example in the working life (*n* = 133)	100	75.2	15	11.3	11	8.3	6	4.5	1	0.8
6. I am self-conscious about my eyes	27	19.7	25	18.2	36	26.3	35	25.5	14	10.2
7. People avoid looking at me because of my eyes (*n* = 136)	72	52.9	33	24.3	21	15.4	9	6.6	1	0.7
8. I feel inferior to others because of my eyes	61	44.5	30	21.9	28	20.4	13	9.5	5	3.6
9. People react differently to me because of my eyes (*n* = 136)	74	54.4	31	22.8	19	14.0	8	5.9	4	2.9
10. I find it hard to initiate contact with people I do not know because of my eyes	76	55.5	22	16.1	15	10.9	18	13.1	6	4.4
AS-20 Functional subscale										
11. I cover or close one eye to see things better	15	10.9	13	9.5	52	38.0	52	38.0	5	3.6
12. I avoid reading because of my eyes	45	32.8	26	19.0	37	27.0	24	17.5	5	3.6
13. I stop doing things because my eyes make it hard to concentrate	30	21.9	36	26.3	50	36.5	19	13.9	2	1.5
14. I have problems with depth perception (*n* = 136)	24	17.6	21	15.4	44	32.4	33	24.3	14	10.3
15. My eyes feel strained	8	5.8	4	2.9	43	31.4	70	51.1	12	8.8
16. I have problems reading because of my eye condition	9	6.6	22	16.1	44	32.1	42	30.7	20	14.6
17. I feel stressed because of my eyes	17	12.4	34	24.8	40	29.2	35	25.5	11	8.0
18. I worry about my eyes	11	8.0	19	13.9	50	36.5	44	32.1	13	9.5
19. I cannot enjoy my hobbies because of my eyes	31	22.6	31	22.6	43	31.4	25	18.2	7	5.1
20. I need to take frequent breaks when reading because of my eyes	27	19.7	31	22.6	28	20.4	42	30.7	9	6.6
Finnish additional items										
21. I find it difficult to go up and down steps	50	36.5	30	21.9	31	22.6	12	8.8	14	10.2
22. I have problems walking on uneven surfaces	41	29.9	27	19.7	32	23.4	25	18.2	12	8.8
23. It is difficult for me to play sports because of my eyes	46	33.6	43	31.4	30	21.9	14	10.2	4	2.9
24. I have problems using mobile devices because of my eyes	32	23.4	41	29.9	34	24.8	24	17.5	6	4.4
Satisfaction with Life Scale, Finnish version [19]	**Fully disagree**		**Partly disagree**		**Neither disagree nor agree**		**Partly agree**		**Fully agree**	
I am satisfied with my life	4	2.9	29	21.2	3	2.2	55	40.1	46	33.6

Note: ^†^ = *n* varies from 133 to 137.

**Table 3:** Descriptive statistics and psychometric properties of the three Finnish AS-20 structures and their subscales (*n* = 137).

	Mean (SD) ^§^		Median (Q1, Q3) ^§^		Cronbach Alfa α^†^	Spearman’s *r* Coefficient ^‡^
AS-20 original (items 1–20)	60.8 (18.0)		63.8 (48.8, 72.5)		0.919	0.501
AS-20 original psychosocial subscale (items 1–10)	70.0 (24.7)		75.0 (53.8, 90.0)		0.953	0.459
AS-20 original functional subscale (items 11–20)	51.6 (19.1)		52.5 (37.5, 66.3)		0.871	0.318
AS-20 with Finnish additions (items 1–24)	61.5 (17.4)		63.5 (52.0, 74.0)		0.923	0.474
Finnish additional items (items 21–24)	65.1 (25.8)		68.8 (50.0, 87.5)		0.858	0.142
	Mean (SD)		Median (Q1, Q3)		Cronbach alfa α ^†^	Spearman’s *r* coefficient ^‡^
AS-20 refined subscales	Clinical use ^§^	Look-up table	Clinical use ^§^	Look-up table		
AS-20 refined, self-perception subscale SP (items 1–4,6)	60.1 (29.0)	62.5 (28.6)	60.0 (37.5, 85,0	63.7 (39.9, 87.5)	0.950	0.439
AS-20 refined -interaction subscale IN (items 5, 7–10)	79.9 (22.9)	79.6 (23.1)	87.5 (65, 100)	89.7 (64.4, 100)	0.905	0.456
AS20 refined, reading function subscale RF (items 12–13,16, 20)	56.3 (25.3)	59.9 (25.3)	50.0 (37.5, 78.1)	57.1 (41.8, 81,0)	0.900	0.194
AS20 refined, general function subscale GF (items 11,15,17–18)	44.9 (18.5)	57.8 (21.0)	43.8 (34.4, 59.4)	59.5 (47.0, 75.8)	0.675	0.344

SD = standard deviation; Q1 = lower quartile; Q3 = upper quartile. Note: ^†^ = *n* varies from 131 to 137; ^‡^ = Spearman correlation coefficient calculated using the item “I am satisfied with my life” from Satisfaction of Life Scale; ^§^ = scores calculated by computing the mean of all completed items.

**Table 4:** The model fit indicators for the three structures of the Finnish AS-20.

Finnish AS-20 Structures and Their Subscales	χ^2^	df	*p*	RMSEA	CFI	TLI	SRMR
AS-20 original (*n* = 131, items 1–20)	628.702	169	*p* < 0.001	0.141	0.949	0.943	0.124
AS-20 with Finnish additions (*n* = 131, items 1–24)	814.509	249	*p* < 0.001	0.129	0.939	0.932	0.121
AS-20 refined (*n* = 132, items 1–13, 15-18, 20)	348.089	129	*p* < 0.001	0.111	0.976	0.971	0.077

χ^2^ = The chi-square goodness of fit; df = degrees of freedom; RMSEA = root mean square error of approximation; CFI = Comparative Fit Index; TLI = Tucker–Lewis Index; SRMR = standardized root mean square residual.

## Data Availability

Due to the ethical approval of this study, the data cannot be shared.

## Supplementary Materials

The following supporting information can be downloaded at: https://www.mdpi.com/article/10.3390/ijerph20042830/s1, the Strengthening the Reporting of Observational Studies in Epidemiology (STROBE) guidelines are attached as Supplementary File S1.
